# TREATMENT OF ADHESIVE CAPSULITIS WITH THE TRIPLE PROCEDURE: INTRA-ARTICULAR INJECTION WITH CORTICOSTEROIDS, HYDRODILATION AND SHOULDER MANIPULATION UNDER SEDATION

**DOI:** 10.1590/1413-785220243206e281046

**Published:** 2025-01-10

**Authors:** Mauro Emilio Conforto Gracitelli, Jorge Henrique Assunção, Micael de Mesquita Paiva, Fernando Brandão de Andrade e Silva, Arnaldo Amado Ferreira, Eduardo Angeli Malavolta

**Affiliations:** 1. Universidade de Sao Paulo, Hospital das Clinicas HC-FMUSP, Faculdade de Medicina, Sao Paulo, SP, Brazil.; 2. Hospital do Coração HCor, Sao Paulo, SP, Brazil.

**Keywords:** Frozen Shoulder, Adhesive Capsulitis of the Shoulder, Manipulation, Intra Articular Injection, Ombro congelado, Capsulite adesiva do ombro, Manipulação, Injeção intra-articular

## Abstract

Objective: There are several conservative treatment options for adhesive capsulitis (AC), but no previous study combines hydrodilation, corticosteroid injection and joint manipulation under sedation (triple procedure), followed by daily home exercises. Methods: Patients included were evaluated before the procedure, at 30 days, 3, 6 and 12 months after treatment in prospective cohort. The outcomes used were the ASES, UCLA, SANE, VAS scales and passive range of motion (ROM). Results: 65 shoulders of 63 patients were included. The mean ASES score progressed from 37.7 ± 17.9 to 94.1 ± 10.3 at 12 months after the procedure (p < 0.001). The mean UCLA went from 15.9 ± 5 to 33.2 ± 2.8 and SANE from 50.4 ± 18.3 to 94.3±9.0. At 12 months, the passive elevation improved from 114°±24° to 176° ± 6°, external rotation in neutral from 29° ± 17° to 72° ± 11° and internal rotation in neutral from 18.6 ± 3.6 points to 9.4 ± 2.4 points. No patient presented a fracture after manipulation. Conclusions: Treatment with the triple procedure resulted in a statistically and clinically relevant improvement in functional results and gains in shoulder range of motion, with no reports of complications. *Level of evidence IV, Prospective study.*

## RESUMO

Objetivo: Há várias opções de tratamento conservador para a capsulite adesiva (CA), mas nenhum estudo anterior combina hidrodilatação, injeção de corticosteroide e manipulação articular sob sedação (procedimento triplo), seguido de exercícios diários em casa. Métodos: Os pacientes incluídos foram avaliados antes do procedimento, 30 dias, 3, 6 e 12 meses após o tratamento em uma coorte prospectiva. Os resultados utilizados foram as escalas ASES, UCLA, SANE, VAS e a amplitude de movimento passivo (ADM). Resultados: Foram incluídos 65 ombros de 63 pacientes. A pontuação média da ASES progrediu de 37,7 ± 17,9 para 94,1 ± 10,3 12 meses após o procedimento (p < 0,001). A UCLA média passou de 15,9 ± 5 para 33,2 ± 2,8 e a SANE de 50,4 ± 18,3 para 94,3 ± 9,0. Em 12 meses, a elevação passiva melhorou de 114° ± 24° para 176° ± 6°, a rotação externa em ponto morto de 29° ± 17° para 72° ± 11° e a rotação interna em ponto morto de 18,6 ± 3,6 pontos para 9,4 ± 2,4 pontos. Nenhum paciente apresentou fratura após a manipulação. Conclusões: O tratamento com o procedimento triplo resultou em uma melhora estatística e clinicamente relevante nos resultados funcionais e ganhos na amplitude de movimento do ombro, sem relatos de complicações. *Nível de evidência IV, Estudo prospectivo.*

## INTRODUCTION

 Adhesive capsulitis is a disease that affects 2 to 5% of the population, ^1^ with a 13.5% prevalence in an outpatient clinic specialized on shoulder. ^2^ Symptoms involve pain, which varies depending on the stage of the disease, and limitation of passive and active movement of the joint. ^3^ ^,^ ^4^ Its cause is unknown, ^5^ and preferentially affects women between 50 and 60 years, ^6^ with increased risk for Asian individuals, ^7^ those with diabetes, ^5^ hypothyroidism ^8^ and dyslipidemia. ^9^

 Several treatment options for adhesive capsulitis exist, with conservative treatment being the preferred option and yielding good results. ^10^ ^,^ ^11^ Among the conservative modalities, physical therapy is the most common modality currently and presents good results in the long term. ^12^ ^,^ ^13^

 The use of intra-articular corticosteroids injections demonstrates benefits, especially in terms of pain improvement, when compared to only rehabilitation. ^13^ ^-^ ^15^ Favejee et al., ^16^ in a systematic review, concluded regarding range of movement (ROM), intra-articular injection associated with physical therapy is more effective than isolated interventions. In contrast, Hopewell et al., ^17^ did not demonstrate clinical differences after 12 months of corticosteroids injection treatment, but observed better results for this group at 2 months of follow-up. 

 Shoulder manipulation under anesthesia is another treatment option. In a randomized study, Kivimaki et al. ^18^ demonstrated better ROM after 3 months, mainly for flexion, but with no difference in pain and function, compared to a control group with specific exercises. And in a current multicenter randomized trial, the authors observed that manipulation associated with corticosteroids injection was the most cost-effective treatment. ^19^

 Capsular hydrodilation is also described, with results superior to placebo for early ROM (3 weeks). ^20^ Buchbinder et al. ^20^ showed that distension followed by physical therapy was more effective compared to physical therapy alone in relation to pain and ROM after 8 weeks. ^20^ When compared to injection with corticosteroids, of three randomized studies, only one demonstrated a significant difference in favor of hydrodilation in relation to ROM. ^20^ According to Maund et al. ^21^ arthrographic distension associated with manipulation under anesthesia presents better results on pain and ROM after 6 months, compared to distension alone. 

Despite several comparative articles published, no study matches hydrodilation, injection with corticosteroids and joint manipulation, followed by daily home exercises for passive shoulder stretching. The authors’ hypothesis is that combined treatment under sedation, called the “triple procedure” is effective and safe for the short and medium term for improvement of ROM pain for adhesive capsulitis.

### Objectives

 Primary objective: to analyze functional results, according to the American Shoulder and Elbow Surgeons Standardized Shoulder Assessment Form (ASES) scale ^22^ at 30 days, 3, 6 and 12 months. 

 Secondary objectives: to evaluate the Modified-University of California at Los Angeles Shoulder Rating Scale (UCLA), ^23^ the Single Assessment Numeric Evaluation (SANE) of patients undergoing conservative treatment of adhesive capsulitis, at 30 days, 3, 6 and 12 months of follow-up and evaluating ROM at the same follow-up times. 

## METHODS

### Study design

Patients with adhesive capsulitis were included in a prospective cohort. The patients were treated by 3 shoulder surgeons from the same institution, members of the Brazilian Society of Shoulder and Elbow Surgery and with more than 15 years of experience.

### Population

 Patients older than 18 years and with clinical diagnosis of adhesive capsulitis, according to Zuckerman and Rockito criteria, ^4^ set by limitation of active and passive shoulder ROM when compared to the contralateral side. We used magnetic nuclear resonance of 1.5 T or higher to corroborate the diagnosis in all cases. 

Patients with secondary limited ROM were not included (complete rotator cuff tear, glenohumeral arthrosis, osteonecrosis of the humeral head, shoulder fractures or dislocation). Patients were also not included if they had bone deformities of the humerus or scapula, previous shoulder surgery, bones and soft tissues tumors, as well as patients with infection in the affected shoulder and the lack of mental capacity to understand the questionnaires. Patients lost to follow-up before the 3-month evaluation were excluded. After evaluating the inclusion criteria, the patients filled out the informed consent form.

### Interventions

The patients underwent, while admitted to a day hospital, the combination of 3 non-surgical techniques: joint injection with corticosteroids, hydrodilation and joint manipulation under sedation (triple procedure).

The patients underwent anesthetic sedation, without the use of a mask or intubation. An anesthetic (5 ml of 2% lidocaine without vasoconstrictor) was applied to the anterior surface of the shoulder, 1 cm lateral to the coracoid, directed towards the joint. Next, a Jelco 16 was used for joint injection of another 5 ml of 2% lidocaine without vasoconstrictor, 10 ml of 7.5 ropivacaine, 40 mg of triamcinolone hexacetonide (2 ml), followed by another 10 to 30 ml of SF 0.9%. In cases of doubt about the joint location, ultrasound or radioscopy was used, at the physician’s discretion.

**Figure 1.**
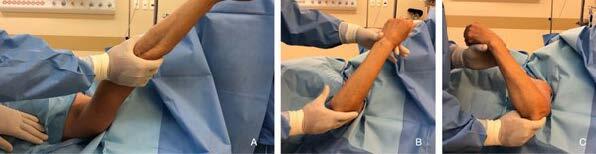
Range of movement before the procedure: flexion (A), lateral rotation (B) and medial rotation (C).

**Figure 2.**
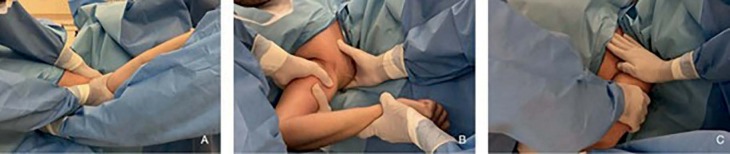
Manipulation under sedation, to gain flexion (A), lateral rotation (B) and medial rotation (C).

**Figure 3.**
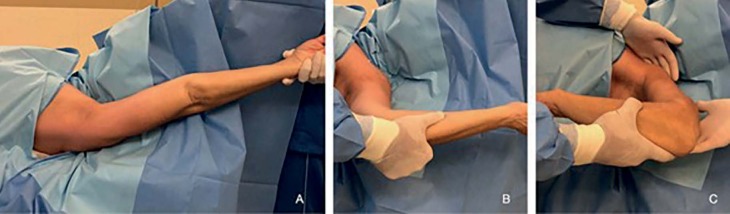
Range of movement immediately after the procedure: flexion (A), lateral rotation (B) and medial rotation (C).

 The patients then underwent shoulder manipulation. We performed progressive movements, with a lever arm close to the shoulder and with stabilization of the scapula and clavicle. Forced elevation was initially performed, followed by abduction, external rotation in abduction, internal rotation in abduction and adduction (Figure 1 , 2 and 3 ). The same procedure was repeated once more to ensure the maximum possible ROM gain. No aggressive manipulation was performed, and if the amplitude did not improve, the procedure was interrupted. 

**Figure 4.**
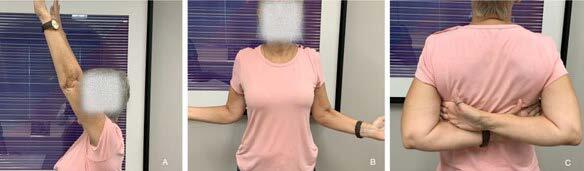
Range of movement 30 days after the procedure: flexion (A), lateral rotation (B) and medial rotation (C).

The patients were discharged 3 hours after the procedure, medicated with dipyrone 1 g 6/6 h for 7 days, non-hormonal anti-inflammatory for 3 days and opioids if severe pain (EVA > 7). Patients received a pamphlet with guidelines for home exercises, being taught in person by the doctor and instructed to perform 4x/day for 30 days.

### Outcomes

The outcomes evaluated were the following scales, carried out before treatment and after inclusion in the study, after 1 month, 3, 6 and 12 months:

- American Shoulder and Elbow Surgeons Standardized Shoulder Assessment Form (ASES) ^22^ (primary outcome)
- Modified University of California at Los Angeles Shoulder Rating Scale (UCLA) ^23^
- Single Assessment Numeric Evaluation (SANE)
- Passive range of motion: assessed with the patient in upright position. The maximum tolerated position was measured by the evaluator. Elevation, lateral and medial rotation of the shoulder at 0 and 90 degrees of abduction (or the greatest tolerated abduction) were evaluated. All measurements were performed with a goniometer, except medial rotation at 0 degrees, which was evaluated according to the position of the patient’s hand in relation to the vertebral spinous processes and, subsequently, converted to continuous numbers, using a scale from 1 to 25 ( T1 to T12 are equivalent to values from 1 to 12, L1 to L5 to values from 13 to 17, the sacrum is equivalent to 20 and the trochanter is equivalent to 25).
- Complications: fractures, rotator cuff tear, shoulder instability

### Variables

The following were patient-related variables were evaluated: age, gender, dominance, ethnicity, thyroid diseases, diabetes, dyslipidemia and history of previous adhesive capsulitis in the contralateral shoulder.

### Sample size

The sample was defined by convenience, picking the total number of patients who wished to participate in the study. The recruitment period was 4 years.

### Bias and follow-up loss

The clinical assessment questionnaires used were applied in person, by telephone or email. For the assessment of ROM, only data collected in person were used. Cases with missing data were treated by imputation with the last observation carried forward (when with a minimum of 2 months of follow-up) or by excluding the patient (when with no post-operative evaluation).

### Statistical analysis

Continuous variables were assessed for normality by the Kolmogorov-Smirnov test, and homogeneity by the Levene test. The continuous data were exposed by mean and standard deviation. Categorical variables were displayed in absolute value and percentage. The score obtained by the different clinical scales and to ROM were compared by Friedman’s Test. Comparison between sequential evaluation times was performed using the Wilcoxon test, with Bonferroni adjustment for multiple comparisons, using p < 0.05 as the significance level. For the primary outcome, a comparative analysis of the results obtained between diabetic and non-diabetic patients was carried out using the Mann-Whitman test. For data analysis, we used the SPSS version 21.0 program (SPSS Inc®, Chicago, IL, USA).

## RESULTS

 During the period evaluated, 72 patients with a confirmed diagnosis of adhesive capsulitis underwent the triple procedure. After applying the inclusion and non-inclusion criteria, our series included 63 patients (65 shoulders). Of these, 46 (69.2%) were female and 32 (49.2%) had the right side treated. The mean age was 51.4 ± 8.8 years. The variables related to patients are exposed in the **Table 1 .**

**Table 1.** Variables related to patients

Variables	N = 65	
Age (years)	51.4	± 8.7
Female	45	(69)
Right Shoulder	32	(49)
Involvement of the dominant arm	31	(48)
Asian Ethnicity	10	(15)
Thyroid diseases	18	(28)
Diabetes	12	(19)
Dyslipidemia	19	(29)
Contralateral capsulitis	7	(11)

Continuous data are presented as means and standard deviations. Categorical data are presented as absolute numbers, with percentages in parentheses.

 According to the ASES, the mean score was 37.7 ± 17.9 in the initial assessment and 90.0 ± 9.6 at 30 days of follow-up, showing significant improvement ( *p* < 0.001). At 12 months of follow-up, the mean score was 94.1 ± 10.3, with statistically significant difference ( *p* < 0,001). The results were also greater than the minimally significant clinical difference in 64 shoulders (98.5% of the sample). The UCLA and SANE functional scales showed significant improvement after the procedure ( *p* < 0.001). Clinical results can be seen in Table 2 . 

 The results regarding ROM are shown in Table 3 . Patients showed significant improvement after the procedure (p < 0.001), as shown by an example in Figure 4 . After 30 days of the procedure, elevation showed a 50° improvement, external rotation at 90° of abduction of 34° and internal rotation at 90° of abduction of 26°. 

No patient had complications related to the procedure, such as fractures or anesthetic complications. No patient underwent arthroscopic capsular release.

**Table 2.** Results of functional scales before and after the procedure.

Scores	Initial	30 days	3 months	6 months	12 months	p
ASES	37.7 ± 17.9 ^*^	90 ± 9.6 ^**^	92.7 ± 9.6	92.1 ± 10.7	94.1 ± 10.3	< 0.001
SANE	50.4 ± 18.3 ^*^	87.6 ± 14.9 ^**^	92 ± 12.7	94 ± 11	94.3 ± 9.0	< 0.001
UCLA	15.9 ± 5 ^*^	32.2 ± 2.9	33.2 ± 2.8	33.1 ± 2.7	33.2 ± 2.8	< 0.001

Data are presented as means and standard deviations.

Post-hoc analysis:

* *p* < 0.005 compared to other post-treatment times (30 days, 3 months, 6 months and 12 months)

** *p* < 0.005 compared to 3 months, 6 months and 12 months

**Table 3.** Range of motion (ROM) results before and after the procedure.

ROM	Initial	30 days	3 months	6 months	12 months	p
Forward elevation	114.3 ± 24.4 ^*^	164.6 ± 14.3 ^**^	174.6 ± 8.1	176.2 ± 6.5	176.5 ± 6	< 0.001
External rotation 0°	29.2 ± 17.2 ^*^	60.9 ± 13.7 ^**^	69.2 ± 12 ^***^	71.2 ± 11.9	72.5 ± 11.5	< 0.001
External rotation 90°	33.1 ± 20.7 ^*^	67.2 ± 16.5 ^**^	77.7 ± 12.7 ^***^	79.2 ± 11.5	80.7 ± 9.9	< 0.001
Internal rotation 0°	18.6 ± 3.6 ^*^	11.8 ± 2.8 ^**^	10 ± 2.9	9.6 ± 2.7	9.4 ± 2.4	< 0.001
Internal rotation 90°	18.9 ± 11.8 ^*^	45.4 ± 14 ^**^	51.6 ± 11.9	53.7 ± 11.7	54.1 ± 11	< 0.001

Data are presented as means and standard deviations.

Post-hoc analysis:

* *p* < 0.005 compared to other post-treatment times (30 days, 3 months, 6 months and 12 months)

** *p* < 0.005 compared to 3 months, 6 months and 12 months

*** *p* < 0.005 compared to 12 months

## DISCUSSION

Our results demonstrate that the treatment of adhesive capsulitis with the triple procedure leads to an early functional improvement, as well as maintenance of the response over 12 months, with satisfactory results demonstrated in all functional scales evaluated.

 Recent studies demonstrate the efficiency of using intra-articular corticosteroids in the treatment of adhesive capsulitis. ^14^ However, short-term assessment is little explored in most studies. ^14^ ^,^ ^24^ Due to the severe symptoms and functional limitations of adhesive capsulitis, short-term responses can significantly impact patient quality of life. In our study, we demonstrated a significant early response in ROM gain and functional pain scales. Although it was not objectively investigated in our study, we were able to observe satisfactory responses within the first 7 days after the procedure. The gain in range of motion was also shown to be rapid and satisfactory 30 days after treatment, with 50° as the mean gain for elevation during the studied period. 

 When comparing functional scales at 12 months, we observed similar results to recently published prospective studies. ^14^ ^,^ ^17^ ^,^ ^19^ ^,^ ^25^ ^,^ ^26^ Few recent studies used 30-day assessment for comparison. When comparing our early results (30 days) to those of the hydrodilation and corticosteroids group by Dai et al., ^25^ we observed superior results regarding elevation (164° versus 105°), external rotation (61° vs 19°) and internal rotation (11 vs 16 points). Our results were also superior to the early results of the same author’s surgical group. We also observed better results regarding the UCLA scale at 3 months (32 vs 22 points). 

 Brealey et al., ^24^ in a pragmatic multicenter randomized study, compared 3 treatment options for adhesive capsulitis: structured physical therapy, manipulation under anesthesia and capsular release by arthroscopy. The authors did not perform hydrodilation in either group. In the first 2 groups, around 80% of cases were concomitantly submitted to intra-articular injection with corticosteroids. The authors demonstrated initial results (3 months of follow-up) that were unfavorable to surgical treatment, but similar results between the groups at 12 months of treatment, with no clinically relevant difference, with slight superiority for the surgical group. However, they demonstrated better cost-effectiveness for the manipulation under anesthesia group. ^27^ Although we do not evaluate the cost of the procedure, we highlight that the combination of a single procedure, in a day hospital and without the use of high-cost medications, associated with performing only home exercises has a great potential for reducing costs for the treatment of adhesive capsulitis. 

 In line with more current studies of manipulation under anesthesia, the complication rate is low and no fractures were observed after the procedure. ^19^ ^,^ ^21^ ^,^ ^28^ ^,^ ^29^ We believe that the absence of complications in our sample is related to how the manipulation was performed. The use of a lever arm very close to the shoulder, both for elevation and rotations, avoided this type of complication. Although it was not objectively evaluated, it was possible to hear the capsular tear in most patients, but in patients with diabetes and older patients, this type of observation was smaller, as was the immediate gain in ROM. Mainly in this subgroup of patients we avoid aggressive manipulation for complete ROM gain. We also believe that hydrodilation may have contributed to the ease of early ROM gain. 

Our study has some limitations. We do not have a control group, which does not allow direct conclusions regarding other treatment options. Our study had a relatively small sample size, but had sufficient power to evaluate the efficacy and safety of the procedure. We did not investigate complications related to the rotator cuff through imaging tests in all cases, and MRI was only performed in patients with any residual symptoms.

As positive points, our study performed a prospective assessment at standardized times, including an early assessment of 30 days. The evaluation of different functional scales and the standardized measurement of ROM is also a strong point of the study. The high short- and medium-term success rate of the triple procedure for adhesive capsulitis will allow us to explore new prospective and randomized studies on this treatment, as well as its comparative cost-effectiveness analysis.

## CONCLUSIONS

Treatment of adhesive capsulitis with the triple procedure (intra-articular corticosteroids injection, hydrodilation and manipulation of the shoulder under sedation) resulted in statistically and clinically relevant improvement in functional results and gains in shoulder range of motion, with no reports of complications.
